# Supplementary material for: STAT5 promotes accessibility and is required for BATF-mediated plasticity at the Il9 locus
Source: Nat Commun. 2020 Sep 28;11:4882. doi: 10.1038/s41467-020-18648-6 (PMC7523001; doi:10.1038/s41467-020-18648-6)
Supplement: Supplementary file 1 — Supplementary Information [file 41467_2020_18648_MOESM1_ESM.pdf]

STAT5 promotes accessibility and is required for BATF-mediated plasticity at the *Il9* locus

Yongyao Fu[1], Jocelyn Wang[1], Gayathri Panangipalli[2], Benjamin J. Ulrich[1], Byunghee Koh[1], Chengxian Xu[3], Rakshin Kharwadkar[4], Xiaona Chu[5], Yue Wang[5], Hongyu Gao[5], Wenting Wu[5], Jie Sun[6], Robert S. Tepper[3], Baohua Zhou[3], Sarath Chandra Janga[2], Kai Yang[3], Mark H. Kaplan[1*]

Supplementary Information

Supplementary Fig. 1 Related to Fig.1

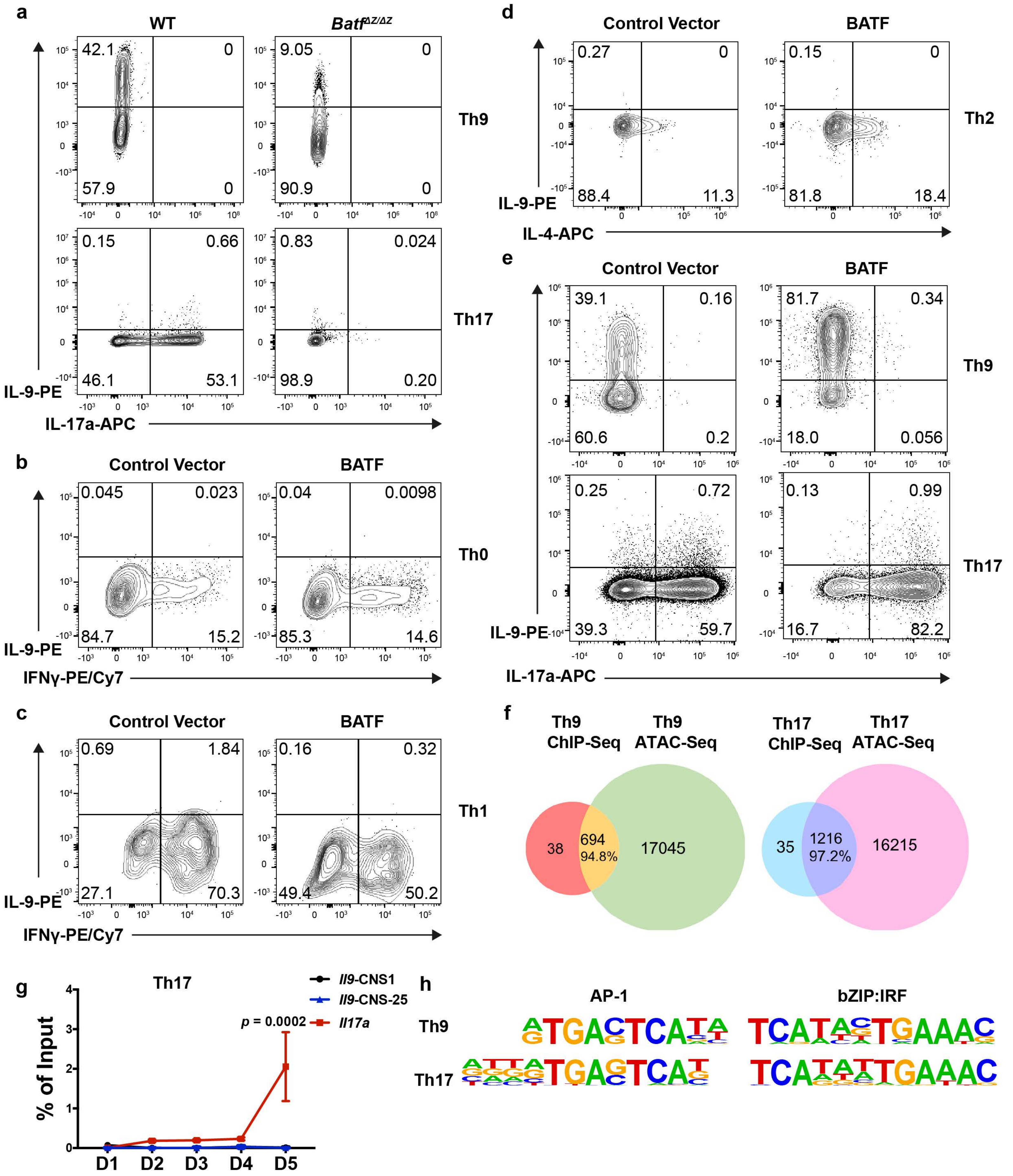

Supplementary Fig. 1. Lineage -specific BATF binding and chromatin structure at the *Il9* gene

Naïve CD4+ T cells were isolated from spleen and differentiated into T helper cells for 5 days. ChIP assay and chromatin accessibility assay were performed on day 5. For cytokine production analysis, Th9 or Th17 cells were stimulated with PMA/Ionomycin for 5 hours, monensin was added for the last 2 hours.

(a) Representative flow cytometric plots of cytokine expression analysis in WT and *Batf*$^{\Delta Z/\Delta Z}$ Th9 and Th17 cells on day 5.

(b-e) Representative flow cytometric plots of cytokine expression analysis in Th0, Th1, Th2, Th9 and Th17 cells transduced with control or BATF expression retrovirus, cells were gated on transduced CD4+ T cells on day 5.

(f) Venn diagram indicating BATF target gene and accessible gene in Th9 and Th17 cells.

(g) Kinetic ChIP analysis of BATF binding on *Il9* locus and *Il17* locus in Th17 cells from D1 to D5 (n=3 per group).

(h) BATF binding motif analysis Th9 and Th17 cells.

Data are mean ± SEM of three mice. The *p* value for **g** is comparison between d1 and d5 Th17 cells. Two-way ANOVA with Sidak's multiple comparisons was used to generate *p* value.

Supplementary Fig. 2 Related to Fig.2

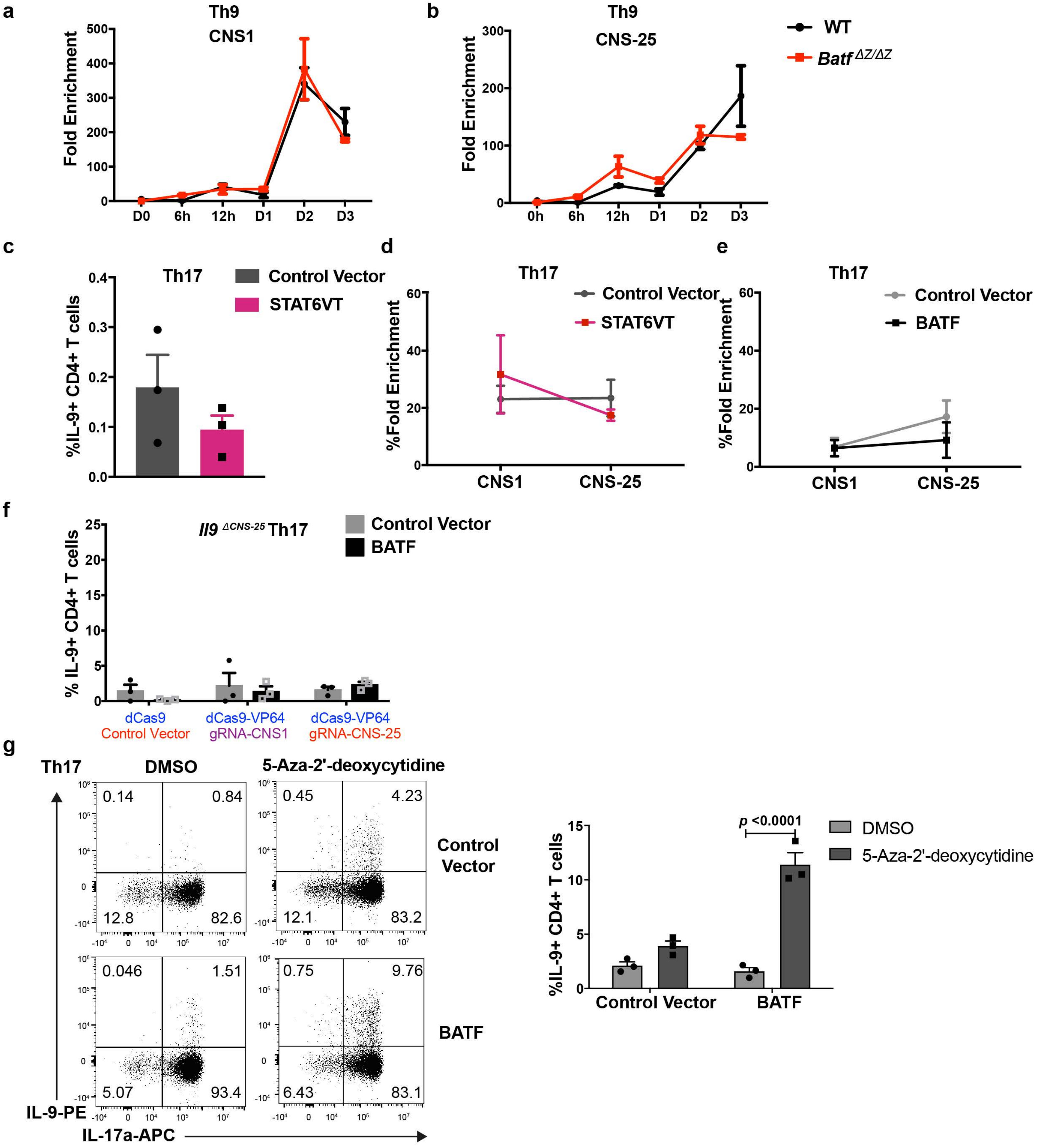

Supplementary Fig. 2. Accessibility is required for BATF to activate *Il9*

Naïve CD4+ T cells were isolated from spleen and differentiated into Th9 and Th17. ChIP assay and chromatin accessibility assay were performed on day 5 or day 6. For cytokine production analysis, Th9 or Th17 cells were stimulated with PMA/Ionomycin for 5 hours, monensin was added for the last 2 hours.

(a-b) Kinetic chromatin accessibility analysis of the *Il9* gene locus in WT or *Batf*$^{\Delta Z/\Delta Z}$ Th9 cells.

(c) Flow cytometric analysis of IL-9 expression in Th17 cells that transduced with control or STAT6VT retrovirus on day1, cells were gated on transduced CD4+ T cells on day 5.

(d) Chromatin accessibility analysis of the *Il9* gene locus in Th17 cells that transduced with control or STAT6VT retrovirus on day 1, transduced cells were sorted on day 5.

(e) Chromatin accessibility analysis of the *Il9* gene locus in Th17 cells transduced with control or BATF retrovirus on day1, transduced cells were sorted on day 5.

(f) dCas9/dCas9-VP64 and gRNA expressing retrovirus was transduced on day 1, BATF vector was transduced on day 4. Flow cytometric analysis of IL-9 expression was analyzed on day 6, cells were gated on transduced CD4+ cells.

(g) Flow cytometric analysis of IL-9 expression in Th17 cells treated with DMSO or 5-Aza-2' deoxycytidine and transduced with control vector or BATF expressing retrovirus on day1, cells were gated on transduced CD4+ T cells on day 5.

Data are mean ± SEM of three mice per experiment. Two-way ANOVA with Sidak's multiple comparisons was used to generate *p* value.

Supplementary Fig. 3 Related to Fig.3

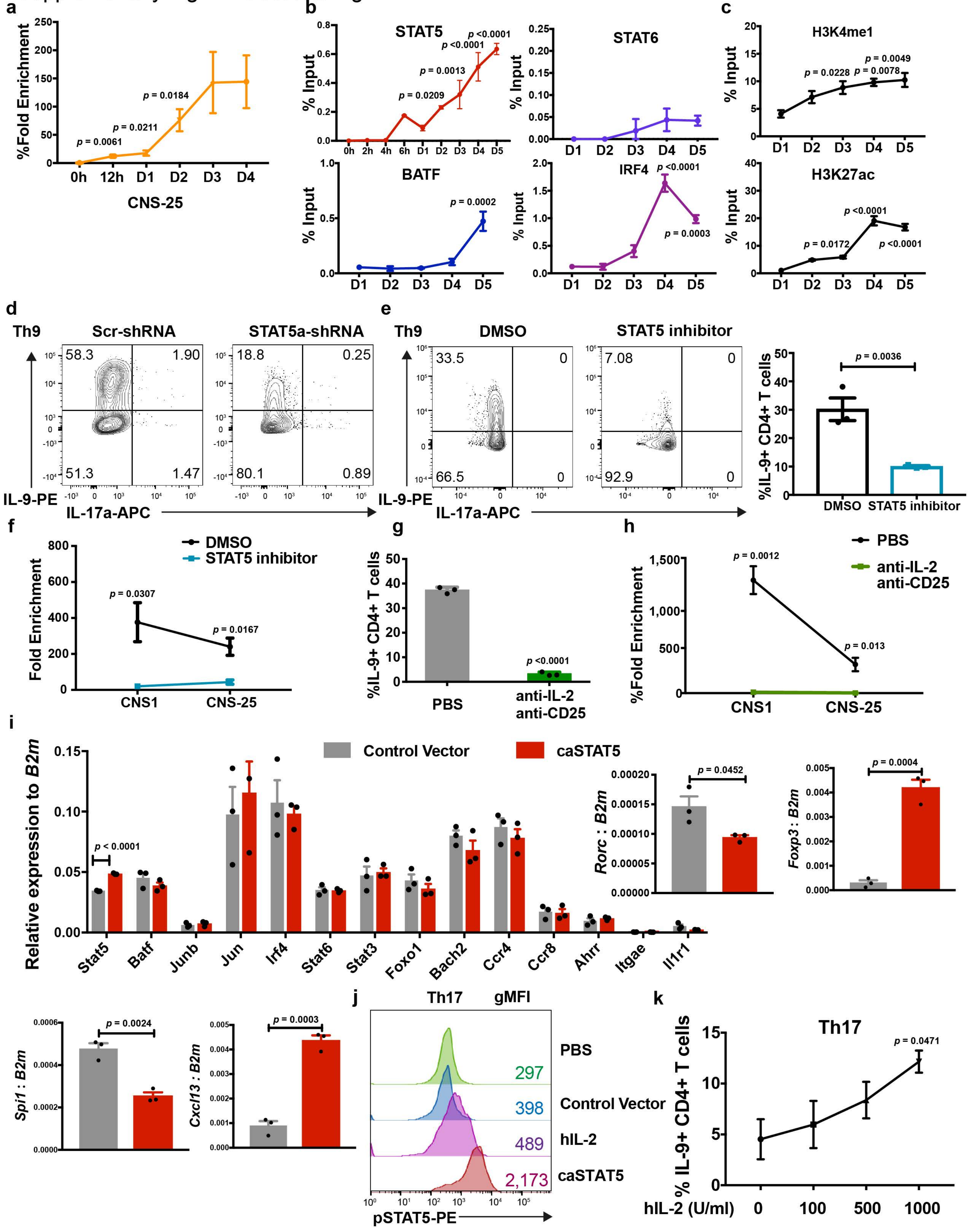

Supplementary Fig. 3. STAT5 regulates *Il9* chromatin accessibility in Th9 cells

Naïve CD4+ T cells were isolated from spleen and differentiated into Th9 and Th17 cells for 5 days. ChIP assay and chromatin accessibility assay were performed on day 5. ShRNA expressing retrovirus was transduced on day 1. For cytokine production analysis, cells were stimulated with PMA/Ionomycin for 5 hours, monensin was added for the last 2 hours.

(a-c) Kinetic analysis of chromatin accessibility, transcription factors and chromatin modification markers binding on *Il9* enhancer locus in Th9 cells during Th9 differentiation.

(d) Representative dot plots of cytokine expression analysis in Th9 cells transduced with control vector or STAT5a-shRNA expressing retrovirus, cells were gated on transduced CD4+ T cells on day 5.

(e-f) Flow cytometric analysis of IL-9 expression and chromatin accessibility analysis of *Il9* gene locus in Th9 cells treated with DMSO or STAT5 inhibitor, cells were analyzed on day 5.

(g-h) Flow cytometric analysis of IL-9 expression and chromatin accessibility analysis Th9 cells treated with PBS or anti-IL-2 and anti-CD25, cells were analyzed on day 5.

(i) mRNA expression analysis by qPCR in Th9 cells transduced with control vector or caSTAT5 expressing vector, transduced cells were sorted on day 5.

(j-k) Histogram of pSTAT5 expression and IL-9 expression in Th17 cells treated with PBS or hIL-2, or transduced with control vector or caSTAT5 expressing vector, cells were analyzed on day 5.

Data are mean ± SEM of three mice per experiment and representative of two independent experiments. The *p* values for **a** and **b** are compared to D0 for chromatin accessibility and STAT5 binding, D1 for other transcription factors and chromatin modification markers binding. One-way ANOVA with a Dunnett's multiple comparison test was used to generate p values for all multiple comparisons in **b,c,k**. Unpaired two-tailed Student t test was used for comparisons in **a, d, f, g, h, i**.

Supplementary Fig. 4 Related to Fig.5

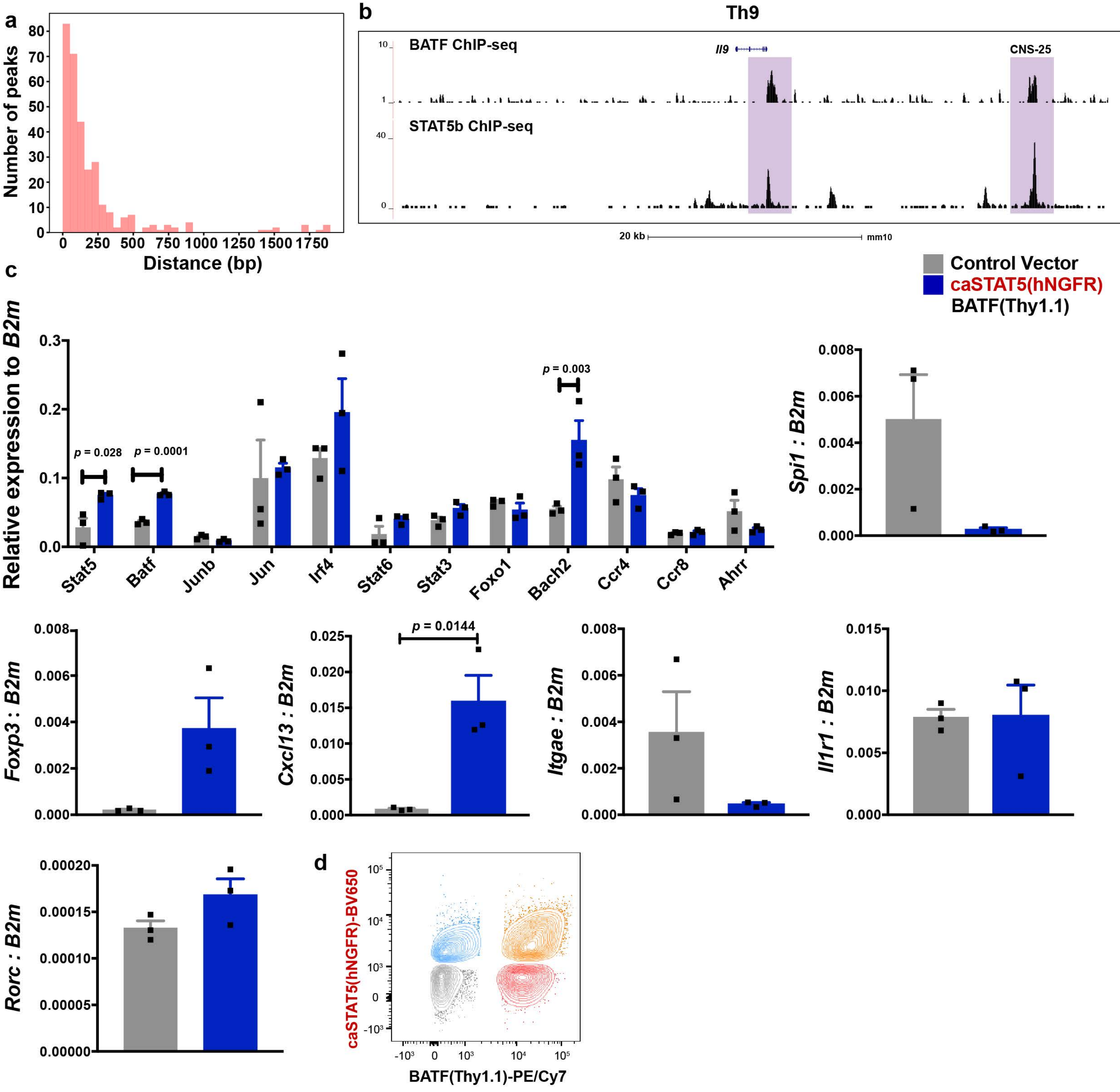

Supplementary Fig. 4. Cooperation between STAT5 and BATF in the plasticity of the *Il9* locus

Naïve CD4+ T cells were isolated from spleen and differentiated into Th lineage for 5 days. Retrovirus expressing caSTAT5 (tagged with hNGFR) and BATF (tagged with Thy1.1) was transduced on day 1.

(a) The distance between BATF binding peaks and STAT5b binding peaks in Th9 cells.

(b) ChIP-seq BATF and STAT5b binding peaks on *Il9* locus in Th9 cells.

(c) mRNA expression analysis by qPCR in Th17 cells co-transduced with control vectors or caSTAT5 and BATF expressing vector, transduced cells were sorted on day 5.

(d) Gating strategy for co-transduced cells in Fig. 4 (b and c).

Data are mean ± SEM from three mice. Unpaired two-tailed Student t-test was used for generating *p* values.

Supplementary Fig. 5 Related to Fig.6

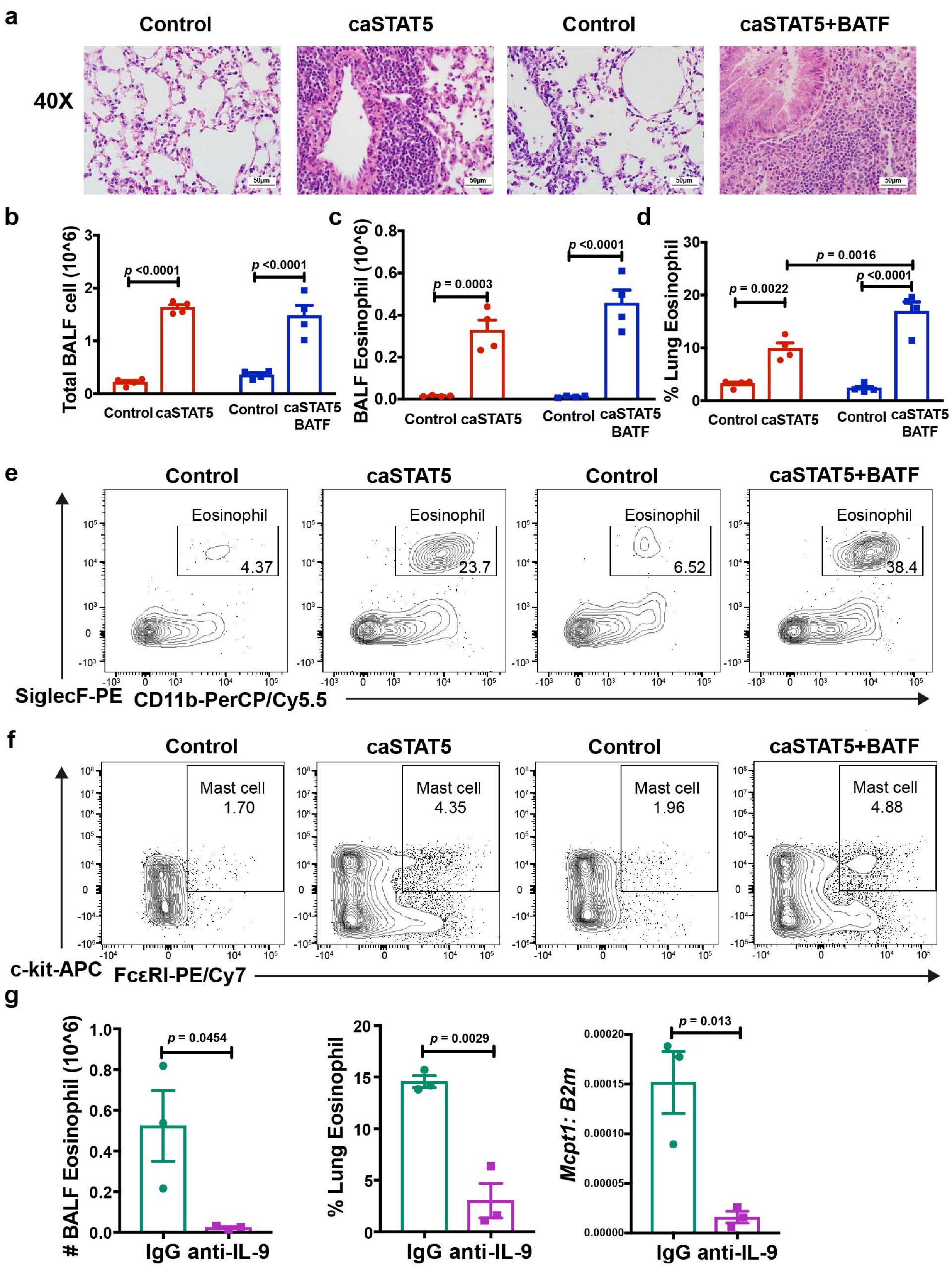

Supplementary Fig. 5. caSTAT5 and BATF convert Th17 cells to a pro-allergic cell population
Cells were cultured and transferred to recipient mice before challenge with OVA as described in Fig. 6.
(a) Lung tissue was stained by H&E staining.
(b) Total BALF cell numbers (n=4 per group).
(c-d) BALF and lung eosinophils were analyzed by flow cytometry (n=4 per group).
(e-f) Representative dot plots of eosinophil and mast cell staining.
(g) BALF and lung eosinophils, and lung *Mcpt1* mRNA expression analysis when IL-9 was blocked in the mice receiving the caSTAT5 and BATF co-transduced Th17 cells (n=3 per group).
Data are mean ± SEM. Two-way ANOVA with Sidak's multiple comparisons was used for multiple comparisons in **b**. Unpaired two-tailed Student t test was used for comparison in **g**.

Supplementary Fig. 6 Related to Fig.7

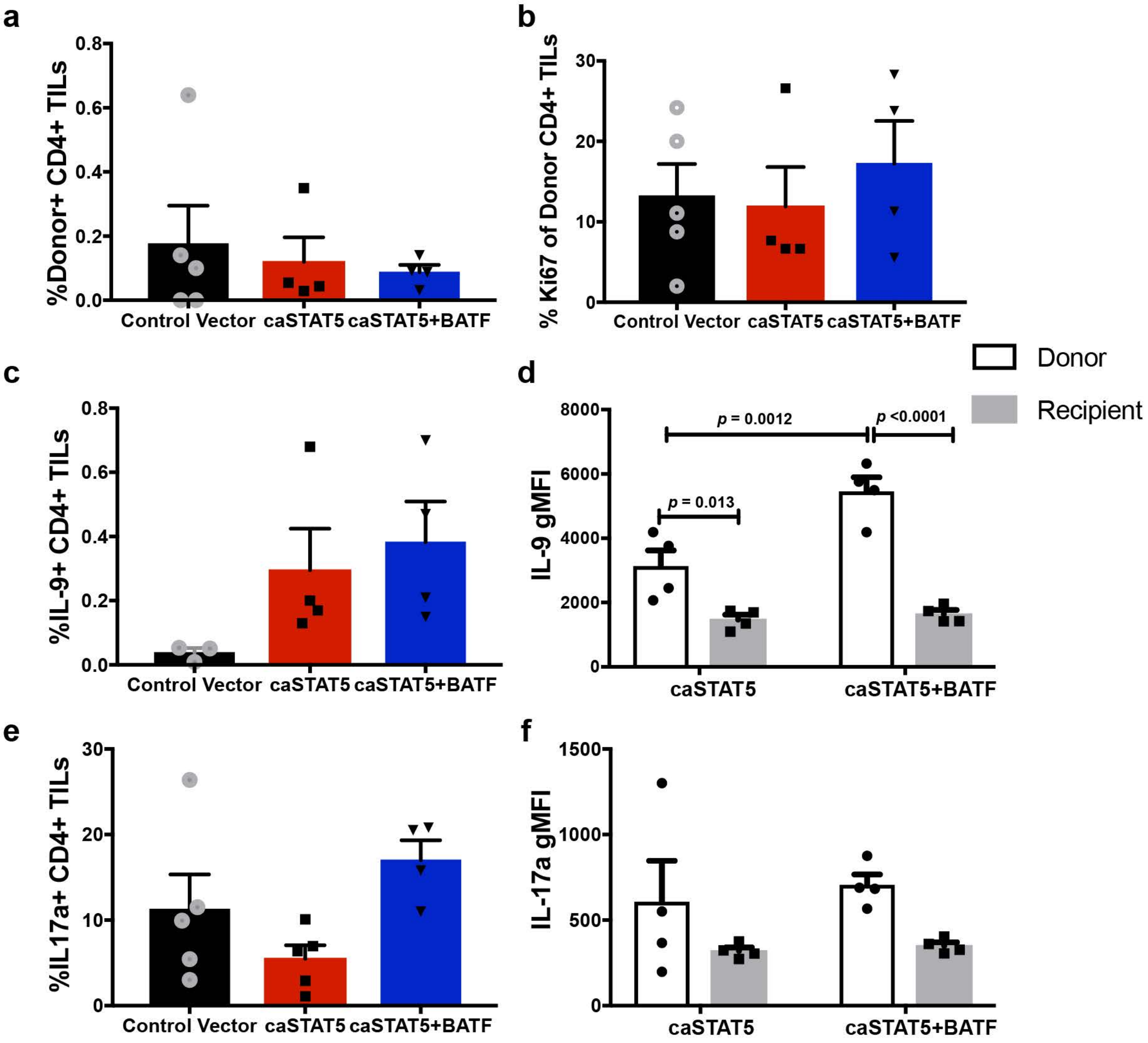

Supplementary Fig. 6. Cooperation between STAT5 and BATF promotes anti-tumor immunity

OT-II CD4 T cells were cultured under Th17 conditions and transduced with control vectors, caSTAT5, or caSTAT5 and BATF retrovirus before transfer to B16-OVA-bearing Boy/J mice as described in figure 7.

(a) Donor CD4+ cells were analyzed by flow cytometry (n=5 for control vector group, n=4 for caSTAT5 and caSTAT5+BATF group).

(b) Ki67+ donor CD4+ cells were analyzed by flow cytometry (n=5 for control vector group, n=4 for caSTAT5 and caSTAT5+BATF group).

(c) IL-9+ CD4+ TILs was analyzed by flow cytometry (n=3 for control vector group, n=4 for caSTAT5 and caSTAT5+BATF group).

(d) IL-9 gMFI was analyzed by flow cytometry, cells are gating on CD4+ T cells (n=4 per group).

(e-f) IL-17a production was analyzed by flow cytometry (n=4 per group).

Data are mean ± SEM. Two-way ANOVA with Sidak's multiple comparisons was used for multiple comparisons.

Supplementary Fig. 7

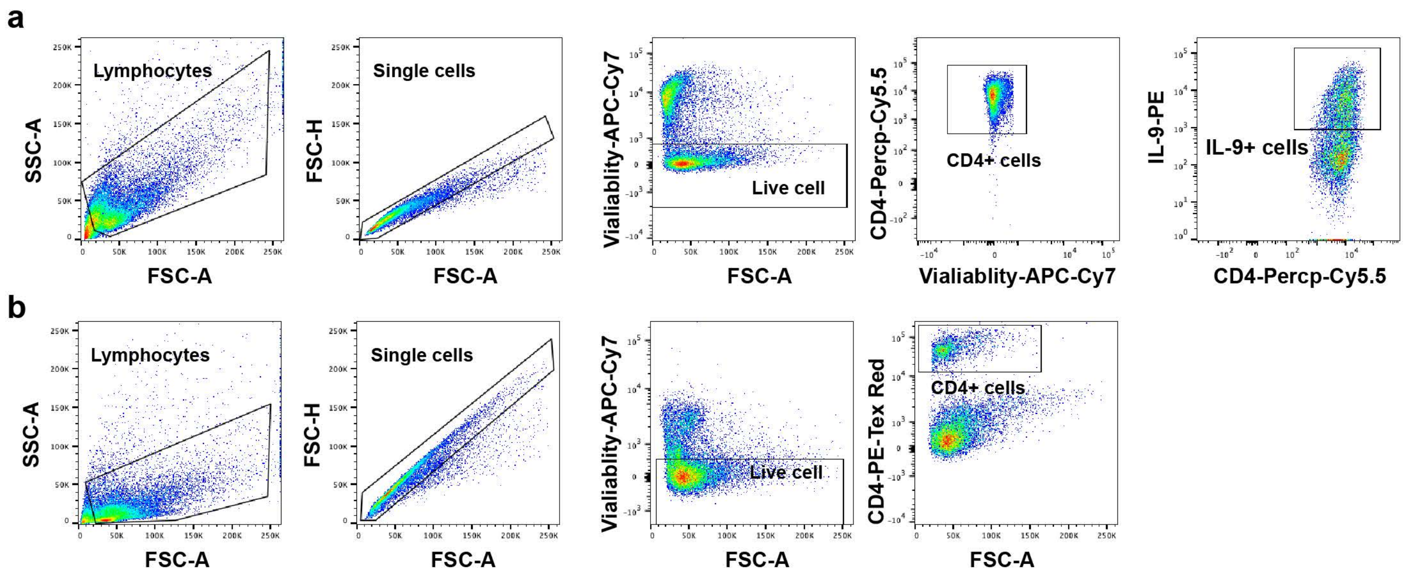

Supplementary Fig. 7. Gating strategy for flow cytometry

Gating strategy for CD4+ T cells in *vitro* (a) and in *vivo* (b).

Supplementary Table 1. Patient sample information

No Asthma	Gender	Weight (kg)	Height (cm)
1	M	20.4	117
2	F	20.2	111.8
3	M	21.9	114
4	M	21.4	115.3
5	M	23	117.5
6	M	21.2	118.4
Asthma Diagnosis			
1	M	18.5	116.5
2	M	19.7	110.5
3	M	24.3	113.3
4	F	20.4	116
5	M	25.3	117.7
6	M	18.5	112.2
7	M	19.4	109.5

Supplementary Table 2. Cytokines and antibodies for Th cell differentiation

Name (Clone)	Company	Catalog Number
anti- Murine CD3 (145-2C11)	BioXCell	BP-0001-1
anti- Murine CD28(37.51)	BioXCell	BE0015-1
anti-mouse IFNγ (XMG1.2)	BioXCell	BE0055
anti-mouse IL-4 (11B11)	BioXCell	BE0045
anti-mouse IL-2 (JES6-5H4)	BioXCell	BE0042
Recombinant Human IL-2	Peprotech	200-02
Recombinant Murine IL-4	Peprotech	214-14
Recombinant Murine IL-6	Peprotech	216-16
Recombinant Murine IL-12 p70	Peprotech	210-12
Human TGF-β1	Miltenyi Biotec	130-095-067
Mouse IL-1β	Miltenyi Biotec	130-101-682
Recombinant Mouse IL-23 Protein	R&D Systems	1887-ML-010
InVivoMAb anti-human IFNγ (B133.5)	BioXCell	BE0235
Recombinant Human IL-4	Peprotech	200-04

Supplementary Table 3. Fluorescent antibodies for flow cytometric analysis

Antigen/Name	Clone	Fluorochrome	Company	Dilution	Catalog Number
Mouse CD11b	M1/70	PerCP-Cy5.5	eBioscience	1:200	45-0112-82
Mouse CD11c	N418	PE-Cy7	eBioscience	1:200	25-0114-82
Mouse CD3	145-2C11	PerCP-Cy5.5	BD Biosciences	1:200	551163
		FITC		1:200	553061
Mouse CD4	GK1.5	FITC	BD Biosciences	1:200	553729
		PE	BioLegend	1:200	100408
		PerCP-Cy5.5	BioLegend	1:200	100434
		PE-Cy7	BioLegend	1:200	100422
		APC	BioLegend	1:200	100412
		APC-Cy7	BD Biosciences	1:200	552051
Mouse CD4	RM4-5	PE-Tex Red	eBioscience	1:200	61-0042-82
Mouse CD49b	HMα2	PE	BioLegend	1:200	103506
Mouse c-Kit	2B8	FITC	BioLegend	1:200	105805
		APC		1:200	105811
Mouse F4/80	BM8	FITC	BioLegend	1:200	123108
Mouse FcεR1	MAR-1	PE-Cy7	BioLegend	1:200	134317
Mouse Foxp3	MF23	FITC	BD Biosciences	1:200	560403
Mouse IFN-γ	XMG 1.2	PerCP-Cy5.5	eBioscience	1:200	45-7311-82
Mouse IL-17A	eBio17B7	PE-Cy7	eBioscience	1:200	25-7177-82
Mouse IL-4	11B11	AF647	BioLegend	1:200	504110
Mouse IL-9	RM9A4	PE	BioLegend	1:200	514104
Mouse IL-13	eBio13A	AF488	eBioscience	1:200	53-7133-82
Mouse IL-5	TRFK5	TRFK5	BD Biosciences	1:200	554396
Mouse Ly6G	1A8	APC	BioLegend	1:400	127613
	RB6-8C5	FITC	BD Biosciences	1:400	553126
Mouse SiglecF	E50-2440	PE	BD Biosciences	1:200	552126
Mouse CD64	X54-5/7.1	FITC	BioLegend	1:200	139316
Mouse Mertk	2B10C42	PE	BioLegend	1:200	151506
Fixable Viability dye		eFluor 780	eBioscience	1:1600	65-0865-14
Human CCR4	L291H4	APC	BioLegend	1:100	359408
Human CD4	A161A1	PE-Cy7	BioLegend	1:200	357410
Human IL-9	MH9A4	PE	BioLegend	1:200	507603

Human IL-17	eBio64DEC17	PerCP-Cy5.5	eBioscience	1:200	45-7179-42

Supplementary Table 4. Taqman probes for qPCR

Gene	Catalog Number
β2m	Mm00437762_m1
β-actin	Mm02619580_g1
Batf	Mm00479410_m1
Stat5	Mm03053818_s1
Junb	Mm04243546_m1
Foxp3	Mm00475165_m1
Jun	Mm07296811_m1
Spi1	Mm00488142_m1
Irf4	Mm00516431_m1
Stat6	Mm01160477_m1
Stat3	Mm01219775_m1
Foxo1	Mm00490671_m1
Bach2	Mm00464379_m1
Ccr4	Mm00438271_m1
Ccr8	Mm99999115_s1
Cxcl13	Mm04214185_s1
Itgae	Mm00434443_m1
Ahrr	Mm00477443_m1
Rorc	Mm00441144_g1
Il1r1	Mm00434237_m1
Mcpt1	Mm00656886_g1

Supplementary Table 5. CRISPR/Cas9 plasmids

Construct	Oligo	Overhang	Protospacer	Overhang
New PX330A_D10A 1X2	sgRNA-1 sense	5'-CACCG	(N)20	
	sgRNA-1 antisense	3'-C	(N)20 complement	CAAA-5'
PX330S_2	sgRNA-1 sense	5'-CACCG	(N)20	
	sgRNA-1 antisense	3'-C	(N)20 complement	CAAA-5'

Supplementary Table 6. Sequences of gRNAs targeting *Il9* promoter and enhancer

gRNAs	Target location	Protospacer sequence (5'-3')
Il9 CNS1	CNS1	CCAACATGTTGACGGGAGTC
Il9 CNS1	CNS1	TTCTCAGAGGTGTATGTACG
Il9 CNS-25	CNS-25	CAATCACCTAGCTAACTCGG
Il9 CNS-25	CNS-25	TGCATTCGAGTCCCCAAATG

Supplementary Table 7. Antibodies for ChIP assay

Antigen/Name	Clone	Host	Company	Dilution	Catalog Number
BATF	D7C5	Rabbit	Cell Signaling Technology	1:50	8638
STAT5	D2O6Y	Rabbit	Cell Signaling Technology	1:50	94025
STAT6	D3H4	Rabbit	Cell Signaling Technology	1:50	5397
H3K27ac	polyclonal	Rabbit	Abcam	1:100	ab4729
H3K27me3	mAbcam6002	mouse	Abcam	1:100	ab6002
H3K4me1	polyclonal	Rabbit	Abcam	1:100	ab8895
H3K4me3	polyclonal	Rabbit	Abcam	1:100	ab8580
RNA Polymerase II	CTD4H8	Mouse	Millipore	1:50	05-623-Z

Normal Rabbit IgG	polyclonal	Rabbit	Millipore	1:200	12-370

Supplementary Table 8. Sequences of ChIP and Chromatin accessibility assay primers

Mouse		
Primers	Forward (5'-3')	Reverse (5'-3')
Il9 promoter -5bp ~ -67bp	GTGGGCACTGGGTATCAGTTTGATGT	CAGTCTACCAGCATCTTCCAGTCTAG
Il9 CNS -25_1	ATGTCATGAGGCTTGTCTGC	ACTCCTAATCTTCAAGCCCCT
Il9 CNS -25_2	AGCAGGCGACCACTTTAAAA	GCCAACTCTCAGCATGTGTT
Il9 -35 kb	GAGGGAGAGGGGAAAACACA	TACCGCTCCGCAGTCTAAAT
Human		
Primers	Forward (5'-3')	Reverse (5'-3')
IL9 promoter	AAGTGGCCCCAACTTACAGA	CGCTTGCAGACACCTTCAAA
IL9 CNS -18_1	ACCTAGCCCACTGTGCAACT	CATGATGACCCTGTGGTCTG
IL9 CNS -18_2	TTTCAGAGTCAGAAGAAAAGATGG	CATTTAGGGTGTTGCCTTTCA

Supplementary Table 9. ELISA capture and biotinylated secondary antibodies

Target	Description	Company	Dilution	Catalog Number
MCPT1	Mouse MCPT-1 uncoated ELISA Kit	Invitrogen	1:250	88-7503-88
IL-9	Mouse IL-9 ELISA MAX Deluxe	BioLegend	1:200	442704

Supplementary Table 10. Key commercial assay

Description	Company	Catalog Number
truChIP Chromatin Shearing Kit with Formaldehyde	Covaris	520154
Illumina Nextera® DNA library preparation kit	Illumina	FC-121-1030
Foxp3 / Transcription Factor Staining Buffer Set	eBioscience	00-5523-00
eBioscience™ Permeabilization Buffer (10X)	eBioscience	00-8333-56
EpiQuik Chromatin Accessibility Assay Kit	EpiGentek	P-1047-48
